# Oncogenic role of lncRNA CRNDE in acute promyelocytic leukemia and *NPM1-*mutant acute myeloid leukemia

**DOI:** 10.1038/s41420-020-00359-y

**Published:** 2020-11-11

**Authors:** Xuefei Ma, Wei Zhang, Ming Zhao, Shufen Li, Wen Jin, Kankan Wang

**Affiliations:** 1 Shanghai Institute of Hematology, State Key Laboratory of Medical Genomics, National Research Center for Translational Medicine, Ruijin Hospital, Shanghai Jiao Tong University School of Medicine, 200025 Shanghai, China; 2 School of Life Sciences and Biotechnology, Shanghai Jiao Tong University, 200240 Shanghai, China; 3 CNRS-LIA Hematology and Cancer, Sino-French Research Center for Life Sciences and Genomics, Ruijin Hospital, Shanghai Jiao Tong University School of Medicine, 200025 Shanghai, China

## Abstract

The PML/RARα fusion protein acts in concert with cooperative genetic events in the development of acute promyelocytic leukemia (APL). However, oncogenic long non-coding RNAs (lncRNAs) cooperating with PML/RARα remain under-explored. Here, we first identified a set of pathogenesis-related lncRNAs, aberrantly expressed in APL using RNA-seq data from a large cohort of acute myeloid leukemia (AML) patients and normal counterparts. Among the pathogenesis-related lncRNAs, one of the evolutionarily conservative lncRNAs CRNDE (Colorectal Neoplasia Differentially Expressed) drew our attention. We found that CRNDE was highly expressed in the disease state but not in the preleukemic stage of APL, suggesting that CRNDE might be a secondary event coordinating with PML/RARα to promote APL development. Functional analysis showed that CRNDE knockdown induced differentiation and inhibited proliferation of APL cells, and prolonged survival of APL mice. Further mechanistic studies showed that CRNDE elicited its oncogenic effects through binding the miR-181 family and thereby regulating *NOTCH2*. Finally, we found that high CRNDE expression was also significantly correlated with *NPM1* mutations and contributed to the differentiation block in *NPM1*-mutant AML. Collectively, our findings shed light on the importance of oncogenic lncRNAs in the development of AML and provide a promising target for AML therapy.

## Introduction

Leukemia development involves a series of complex and multifactorial events that lead to dysregulation of genes involved in differentiation, proliferation, and apoptosis of leukemia cells. Extensive studies have focused on the roles of protein-coding genes, which constitute two percent of the human genome, in the initiation, maintenance, and development of leukemia^1,2^. Recent rapid progress in RNA sequencing has revealed that non-coding RNA (ncRNA), especially long non-coding RNAs (lncRNA) that are three times (nearly 60,000 lncRNA genes) more abundant than protein-coding genes, have critical regulatory roles in malignant transformation and progression^3^. However, only a few lncRNAs have been functionally and mechanically characterized in the development and progression of leukemia. Identifying lncRNAs with oncogenic or tumor-suppressive properties is thus crucial for deciphering the mechanisms of leukemia development and finding new strategies for the treatment of leukemia^4^.

Acute promyelocytic leukemia (APL) is characterized by a typical t(15; 17)(q22; q21) translocation, which generates a fusion gene *PML/RARα* between the *Promyelocytic Leukemia* (*PML*) gene and the *Retinoic Acid Receptor α* (*RARα*) gene. Intensive studies have been made to elucidate the roles of PML/RARα in malignant transformation and differentiation block at the promyelocytic stage of myelopoiesis^5^. However, based on murine APL models, it appears that PML/RARα alone is not sufficient to cause leukemia, and cooperating events are required for the development of APL. Co-expression of Bcl2 and PML/RARα, for example, can more rapidly induce leukemia development in mice than expressing PML/RARα alone^6^. Also, the co-existence of amplified MYC or activated FLT3 with PML/RARα can promote the transformation of APL in mice^7,8^. However, most of our knowledge about the cooperating events during APL development has come from the studies conducted on protein-coding genes, and the function of lncRNAs involved in APL leukemogenesis is still largely unknown.

In this study, we identified a series of pathogenesis-related lncRNAs in APL by comparing a large number of RNA-seq data from acute myeloid leukemia (AML) and normal bone marrows. Among the dysregulated lncRNAs, we characterized lncRNA CRNDE (Colorectal Neoplasia Differentially Expressed), which played an oncogenic role in promoting APL progression in vitro and in vivo. CRNDE could directly bind to the miR-181 family and thus regulating *NOTCH2*. Moreover, high CRNDE expression was also found in *NPM1*-mutant AML through influencing differentiation. Our results demonstrate the importance of lncRNAs involved in leukemogenesis and provide a clinical therapeutic target for AML.

## Results

### Identification of pathogenesis-related lncRNAs in APL

**Fig. 1:**
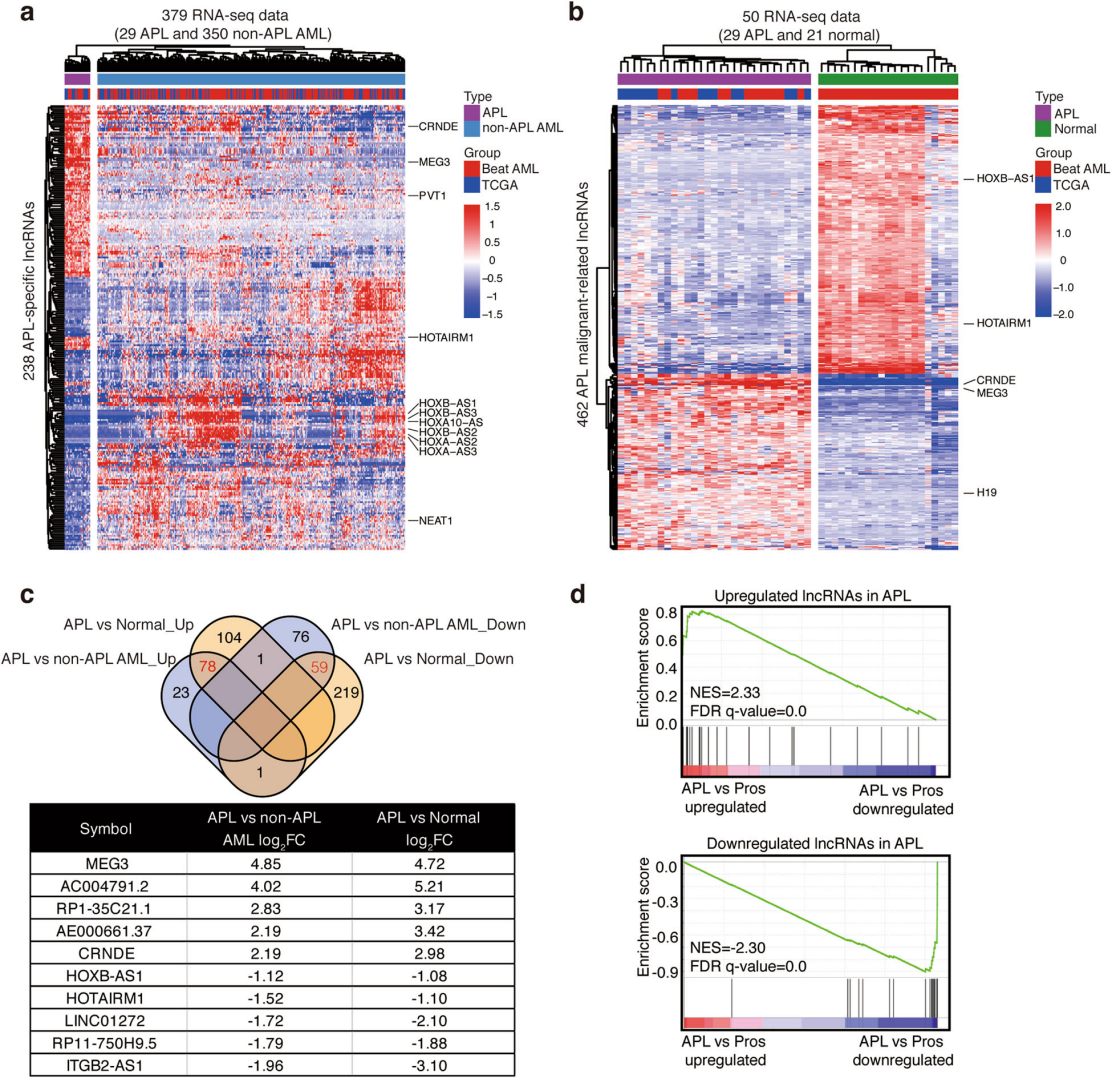
Identification of pathogenesis-related lncRNAs in APL. **a**–**b** Heatmap displays the expression values of differentially expressed lncRNAs between APL and non-APL AML (**a**) or APL and normal bone marrows (**b**). The RNA-seq data of 29 APL, 350 non-APL AML and 21 normal bone marrows (Normal) were retrieved from the TCGA and Beat AML cohorts. The 102 upregulated and 136 downregulated lncRNAs clearly separate APL from non-APL AML (**a**). The 183 upregulated and 279 downregulated lncRNAs clearly separate APL from normal bone marrows (**b**). **c** Venn diagram shows the overlapped differentially expressed lncRNAs between APL versus non-APL AML (blue) and APL versus normal bone marrows (yellow). Numbers in red represent overlapped lncRNAs upregulated or downregulated in APL. The representative lncRNAs are shown in the table below. **d** Gene set enrichment analysis (GSEA) plots show enrichment for pathogenesis-related lncRNAs upregulated (upper panel) or downregulated (lower panel) in APL versus normal promyelocytes (GSE12662). Pros, promyelocytes; NES, normalized enrichment score; FDR *q*-value, false discovery rate *q*-value.

We first retrieved RNA-seq data of 379 de novo AML samples (29 APL and 350 non-APL AML) and 21 normal counterparts from two cohorts of clinical AML patients (details in “Materials and methods” section) to explore lncRNAs that may contribute to the pathogenesis of APL. First, by comparing APL with non-APL AML, we identified 238 APL-specific lncRNAs (102 upregulated and 136 downregulated), which could clearly distinguish APL from non-APL AML (Fig. 1a, Supplementary Table S1). These suggested that APL had a unique lncRNA expression pattern. Next, we compared the lncRNA profiling of APL compared with normal bone marrow samples and obtained 462 malignant lncRNAs (183 upregulated and 279 downregulated) (Fig. 1b, Supplementary Table S2). Finally, by merging APL-specific and malignant-related lncRNAs, we totally obtained 137 lncRNAs (78 upregulated and 59 downregulated) that had the oncogenic potential in APL development, defined as pathogenesis-related lncRNAs in APL (Fig. 1c, Supplementary Table S3). The importance of these lncRNAs was also validated using transcriptome data of APL versus normal promyelocytes (GSE12662^9^) (Fig. 1d). These pathogenesis-related lncRNAs included some already known to be involved in APL. For example, HOTAIRM1 has been reported to contribute to myeloid differentiation, especially ATRA-induced differentiation of APL cells^10,11^. High expression of MEG3 has been observed in APL patients and regulated miRNA expression in APL^12^. Furthermore, we also found some lncRNAs known to be involved in many types of solid tumors but without clear roles defined in APL, such as CRNDE^13–16^ and ITGB2-AS1^17^.

### Oncogenic lncRNA CRNDE coordinates with PML/RARα in promoting leukemogenesis in APL

**Fig. 2:**
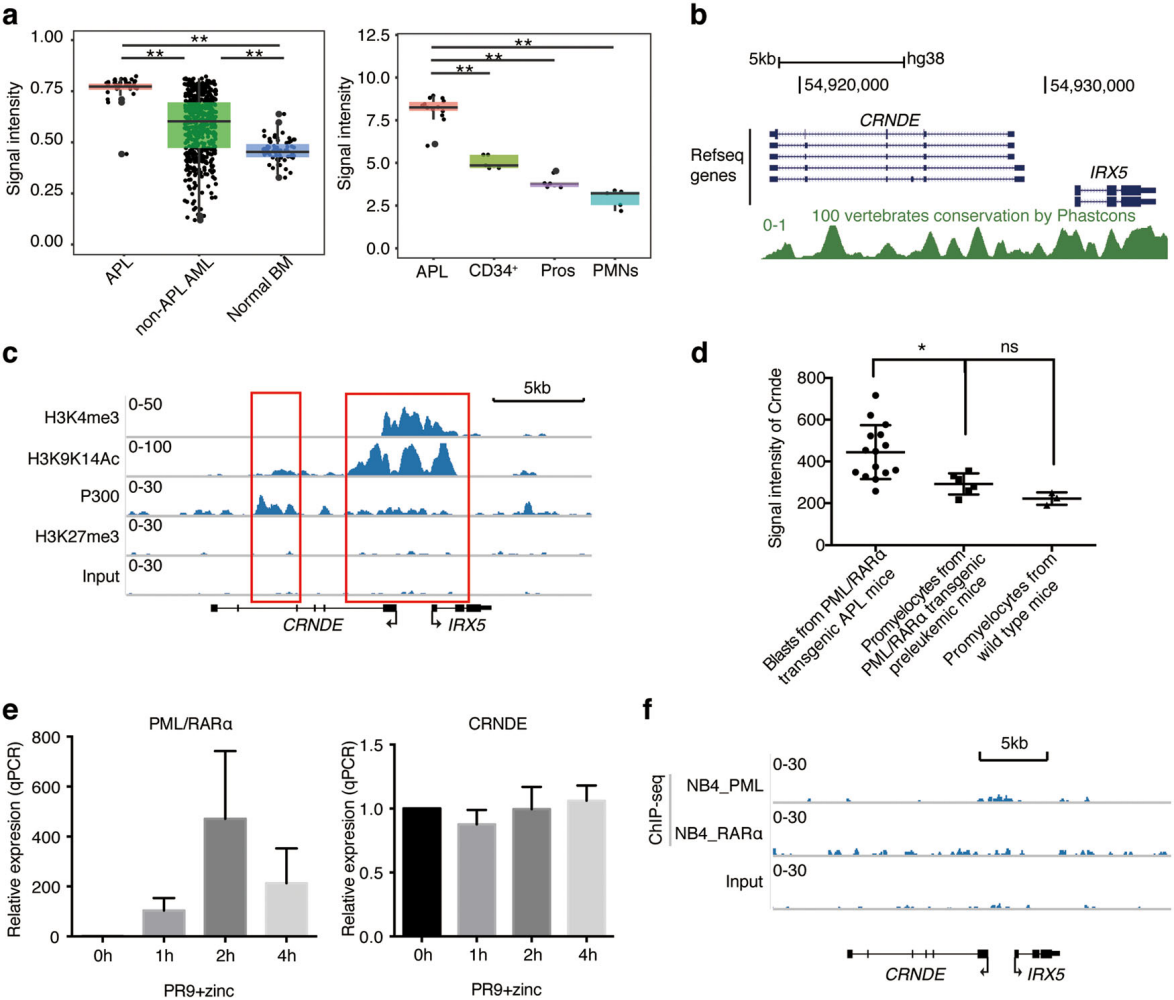
The oncogenic CRNDE cooperates with PML/RARα in promoting APL progression. **a** Comparison of CRNDE expression among APL, non-APL AML and normal counterparts. Left panel shows the signal intensities of CRNDE expression in 37 APL, 505 non-APL AML, and 74 normal bone marrow (BM) samples (GSE13159). Right panel shows the signal intensities of CRNDE expressions in 14 APL, 5 CD34^+^ hematopoietic stem cells specimens (CD34^+^), 5 normal promyelocytes specimens (Pros), and 5 polymorphonuclear neutrophils specimens (PMNs) (GSE12662). Asterisks (**) indicates *P*-value < 0.01. **b** Screenshot from the UCSC genome browser showing the evolutionary conservation within the *CRNDE* locus determined by Phastcons scores. Plotting scale and nucleotide numbering (top) are for chromosome 16, hg38. **c** H3K4me3, H3K9K14Ac, H3K27me3, and P300 ChIP-seq tracks at the *CRNDE* locus in APL cell line NB4 cells (GSE18886) are portrayed using the IGV v1.5 software. Input as a negative control. The arrows indicate transcriptional direction. **d** The signal intensities of Crnde (GSE40022) are presented by the scatter plot in blasts from PML/RARα transgenic APL mice, promyelocytes from PML/RARα transgenic preleukemic mice and wide-type mice. **e** Detection of PML/RARα (left panel) and CRNDE (right panel) expressions in U937-PR9 cells after the treatment of ZnSO4 by qRT-PCR assays. U937-PR9 cells were treated with 100 μM ZnSO_4_ for 0, 1, 2, 4 h. The relative expression was normalized to GAPDH. Data represent the mean ± s.d. from three replicates. **f** Schematic diagram shows that there is no PML or RARα binding signals (GSE18886) within the *CRNDE* gene region in NB4 cells. Input as a negative control.

Next, we were interested in investigating the biological function of pathogenesis-related lncRNAs in APL. CRNDE drew our interest because of the following characteristics. First, CRNDE expression was remarkedly higher in APL samples, as compared with non-APL AML (GSE13159^18,19^), as well as normal counterparts, including normal promyelocytes, CD34^+^ hematopoietic stem cells and polymorphonuclear neutrophils (GSE12662^9^) (Fig. 2a). Second, CRNDE exhibited a high degree of sequence conservation in vertebrates (Fig. 2b), suggesting its functional importance. Third, the epigenetic status of the *CRNDE* locus in APL cells showed a high level of active histone marks (GSE18886^20^) (Fig. 2c), such as H3K4 trimethylation and H3K9K14 acetylation, and an enriched binding of histone acetyltransferase P300, along with the loss of repressive marks, such as H3K9me3. These suggest that CRNDE is activated in APL cells, whose high degree of conservation indicates a vital function in vertebrates.

More importantly, we found that CRNDE was only highly expressed in the disease state of APL but not in the preleukemic stage of APL (Fig. 2d). Previous studies have shown that PML/RARα is necessary but not sufficient to cause leukemia, and cooperating events are required for the development of APL^7,8^. PML/RARα transgenic murine APL models mimic this notion well, in which PML/RARα only does not induce leukemia (PML/RARα transgenic preleukemic mice) and additional secondary events cooperating with PML/RARα promote APL development (PML/RARα transgenic APL mice). We retrieved mouse microarray data (GSE40022^21^) on promyelocytes from PML/RARα transgenic preleukemic mice (no APL occurred) and blasts from PML/RARα transgenic APL mice (APL occurred), as well as normal promyelocytes from wild type mice as control. We found that CRNDE expression was only significantly increased after APL occurred but was not induced in the promyelocytes from PML/RARα transgenic preleukemic mice, as compared with wild type mice (Fig. 2d). These indicated that the oncogenic potential of CRNDE was independent of PML/RARα and CRNDE might coordinate with PML/RARα to exacerbate leukemogenesis in APL. To verify this finding, we detected the expression of CRNDE in the PML/RARα-inducible model U937-PR9 cells and found that CRNDE expression remained unchanged upon PML/RARα induction after ZnSO_4_ treatment (Fig. 2e). Moreover, detailed inspection of chromatin immunoprecipitation sequencing (ChIP-seq) data on the *CRNDE* locus indeed supported that there was no enrichment of PML or RARα binding from 50 Kb upstream or downstream of CRNDE (GSE18886^20^) (Fig. 2f). The above characteristics showed that highly expressed CRNDE was an oncogenic lncRNA through a PML/RARα-independent regulation mechanism, and CRNDE might be a cooperative factor during APL leukemogenesis.

### CRNDE knockdown induces differentiation and inhibits proliferation of APL cells and represses APL progression in vivo

**Fig. 3:**
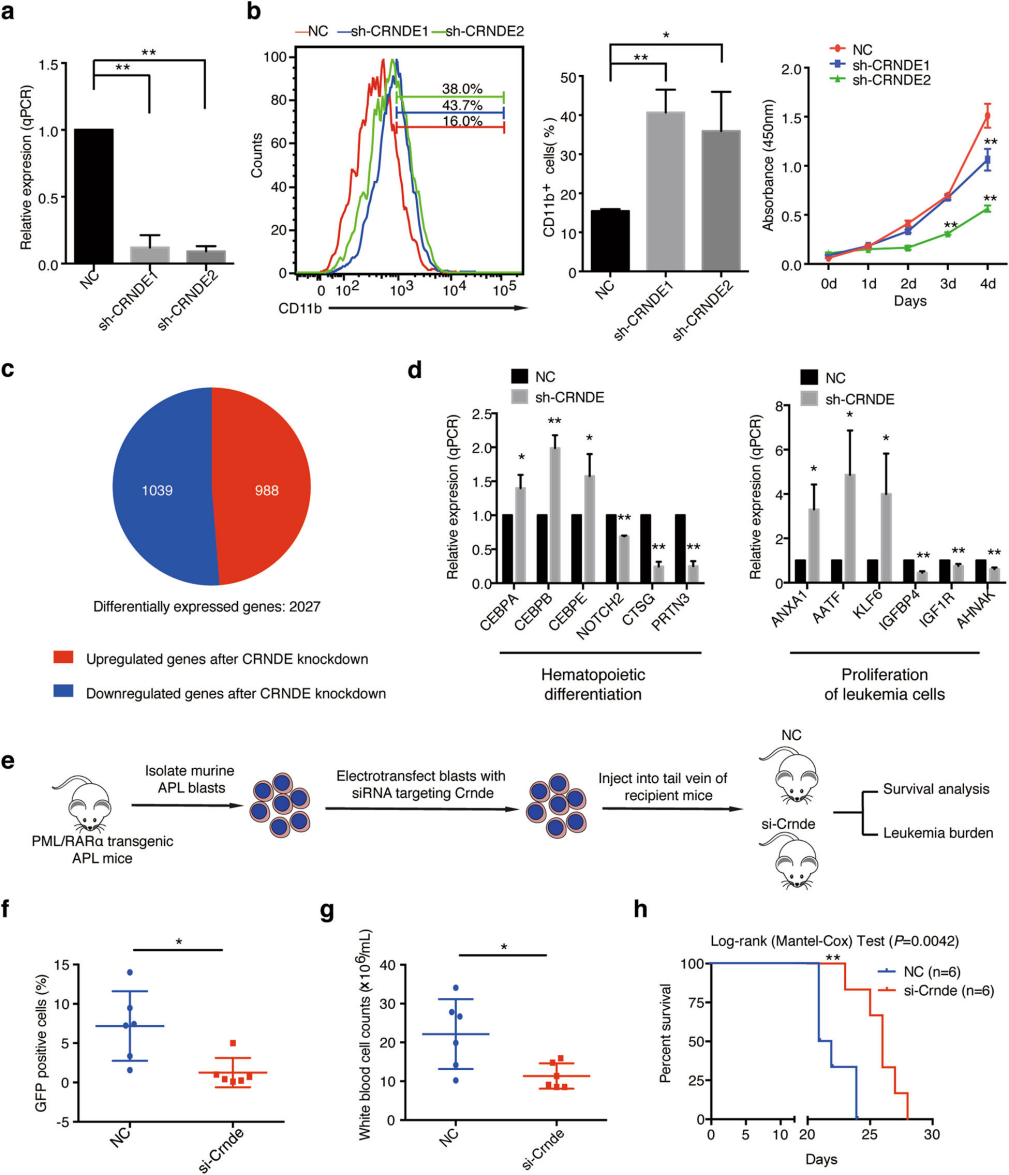
CRNDE knockdown induces differentiation and inhibits proliferation in vitro and suppresses APL leukemogenesis in vivo. **a** The knockdown efficiency of CRNDE was detected in NB4 cells by qRT-PCR after the transfection of CRNDE shRNAs for 72 h. The expression level of GAPDH was used for normalization of qRT-PCR data, and bars represent the mean (s.d.) of three independent experiments. **P < 0.01. **b** Functional analysis was performed after CRNDE knockdown in NB4 cells. Differentiation of NB4 cells was determined by flow cytometric analysis of CD11b expression after transfection with indicated shRNAs, and flow cytometric data was displayed as histogram (left panel) and bar graph (middle panel). The bar graph shows the average percentage of CD11b-positive cells derived from three independent experiments, and data are represented as the mean ± s.d. The proliferation of NB4 cells was detected by CCK8 cell viability assays after transfection with shRNAs at the indicated time (right panel). *P < 0.05, **P < 0.01. **c** Pie chart presents the proportion of differentially expressed genes (fold change > 1.5) based on RNA-seq data after the transfection of CRNDE shRNA for 72 h. **d** qRT-PCR assays were performed to validate differentially expressed genes after CRNDE knockdown in NB4 cells. *P < 0.05, **P < 0.01. **e** The schematic plot represents the procedure performed in vivo. The leukemic blasts from PML/RARα transgenic APL mice were electrotransfected with siRNA targeting Crnde, and then injected into tail vein of the recipient FVB/NJ mice. **f**–**g** The leukemia burden was detected in the peripheral blood of PML/RARα transgenic APL mice with or without Crnde knockdown. (**f**) shows the total white blood cell counts, and (**g**) represents the percentage of GFP-positive APL cells. *P < 0.05. **h** Kaplan–Meier curve shows the survival rates of mice receiving APL blasts transfected with irrelevant control (NC) or siRNA targeting Crnde (*n* = 6 for each model). *P*-value was analyzed by a log-rank (Mantel–Cox) test. **P < 0.01.

To elucidate the biological function of CRNDE in APL, we used shRNA-mediated knockdown approach to stably downregulate CRNDE expression in the APL cell line NB4. We first updated the possible variants using the latest human NCBI AceView database and showed that the *CRNDE* locus produced 12 different transcript variants (Supplementary Fig. S1a). We performed primer-specific qRT-PCR assays to validate the expression of transcript variants of CRNDE and found that CRNDE-g was the most abundant transcript in NB4 (Supplementary Fig. S1b). The following experiments thus focused on CRNDE-g. Two shRNA sequences (sh-CRNDE1 and sh-CRNDE2) efficiently attenuated the expression level of CRNDE (Fig. 3a). By detecting the expression of CD11b, a conventional marker for assessing neutrophilic differentiation, we found CRNDE knockdown induced NB4 cellular differentiation compared with control cells (Fig. 3b). We also detected the influence of CRNDE on NB4 cell proliferation using CCK-8 assays. As a result, the repression of CRNDE significantly inhibited cell proliferation (Fig. 3b).

To mechanistically clarify the functional finding of CRNDE in regulating cell differentiation and proliferation, we performed RNA sequencing to examine transcriptomic changes caused by CRNDE inhibition. A total of 2027 differentially expressed genes were identified (Fig. 3c, Supplementary Table S4). The Ingenuity Pathway Analysis (IPA) analysis indicated that the differentially expressed genes were involved in hematologic system development and function, cellular growth and proliferation, cell death and survival (Table 1, Supplementary Table S5). The differentiation in NB4 cells after CRNDE knockdown might be due to the regulation of hematopoietic differentiation-related genes, such as myeloid-specific markers (*CTSG*, *PRTN3*), hematopoietic transcription factors (*CEBPA*, *CEBPB*, *CEBPE*), and hematopoietic malignant-associated gene *NOTCH2* (Fig. 3d and Table 1). We also noted that some proliferation-associated genes in tumor cells were also significantly regulated by CRNDE knockdown, including *IGFBP4*, *ANXA1*, *KLF6*, *AATF*, and *IFI16* (Table 1). In terms of cell death and survival, several apoptosis-associated genes and pathways were also regulated by CRNDE knockdown. For instance, the members of the BCL2 protein family (*BBC3*, *BCL2L11*, *BAK1*) and MAPK signaling pathway (*MAPK7*, *MAP2K4*) were repressed after CRNDE silencing (Table 1, Supplementary Table S5). The expression of representative differentially expressed genes was further verified by qRT-PCR in CRNDE knockdown cells (Fig. 3d). These data suggested a critical role for CRNDE in blocking differentiation and accelerating proliferation in APL.

**Table 1:** The diseases and functions associated with genes by CRNDE knockdown. (*P* < 0.05).

Enriched biofunction	# of genes	Genes^a^
Hematological disease, hematological system development and function		
Differentiation of hematopoietic progenitor cells	21	CEBPE, CEBPA, GATA2, JUNB, IRF1
Activation, movement and chemotaxis of peripheral blood neutrophils or monocytes	8	ANXA1, CTSG, CCL5, CCR1, ADAM17
Myelopoiesis of hematopoietic progenitor cells	10	HIST1H4A, HIST2H4A, HIST2H4B, STAT5B, IRF1
Cellular growth and proliferation		
Proliferation of leukemia and tumor cell lines	239	IGFBP4, ANXA1, KLF6, AATF, MYC, CDK2, CEBPB, IFI16
Cell death and survival		
Apoptosis of cancer cell and tumor cell lines	183	BBC3, BCL2A1, BCL2L11, BAK1, STAT5A, BIRC2, TP53
Cell death of bone cancer and tumor cell lines	227	KLF9, MAPK7, IGF1R, IRF5, HOXA1, CDK2, CDK9
Cell viability of tumor cell lines	121	BCL2A1, TGFB1, IGF1R, IGF2R, TNFSF13B

^a^Part of CRNDE knockdown-affected genes in the enriched biofunctions.

We further addressed the in vivo effect of CRNDE repression using a transplantable murine APL model. The leukemic blasts from PML/RARα transgenic APL mice were electrotransfected with siRNA targeting Crnde, and then injected into tail vein of the recipient FVB/NJ mice (Fig. 3e). Leukemia engraftment was determined by flow cytometry 19 days after transplantation. As shown in Fig. 3f, compared with negative control mice, si-Crnde-treated mice exhibited a lower percentage of GFP-positive leukemic cells in the peripheral blood. Notably, the white blood cell counts were fewer in si-Crnde-treated mice than those from control mice (Fig. 3g). Moreover, Crnde knockdown significantly extended the survival of the recipients (Fig. 3h). These results showed that Crnde knockdown repressed APL progression in vivo.

### CRNDE elicits its oncogenic effects through direct binding to the miR-181 family

**Fig. 4:**
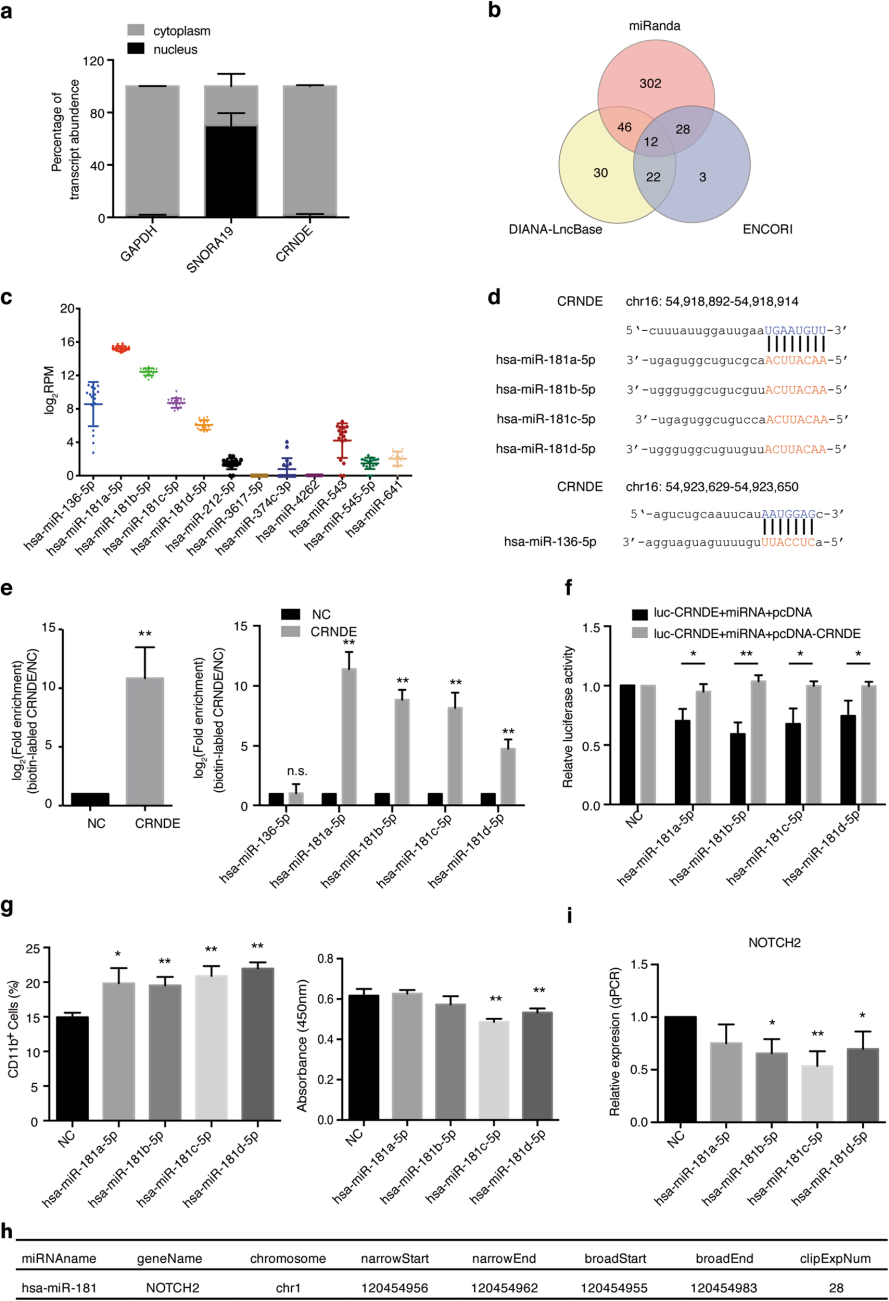
CRNDE exerts its oncogenic effects by sponging miR-181. **a** Subcellular localization of CRNDE in NB4 cells was assessed by the cellular fractionation assay. GAPDH was used as a cytoplasmic control and SNORA19 as a nuclear control. **b** The target miRNAs of CRNDE were predicted by miRanda, DIANA-LncBase version 2 and ENCORI algorithms. **c** Scatter plot representing the log_2_RPM expression values of 12 predicted miRNAs in 17 APL patient samples from the TCGA database. RPM, reads per million. **d** The prediction of CRNDE binding sites on four miR-181 family members and miR-136-5p. The capital letter nucleotides are the seed sequences. **e** In vivo binding of CRNDE and its potential target miRNAs was analyzed by RAP assays in NB4 cells. The expression levels of CRNDE (left panel), and 4 members of the miR-181 family, miR-136 (right panel) were detected by qRT-PCR in RNA samples precipitated with CRNDE-specific oligonucleotide probes from lysates of fixed NB4 cells. An irrelevant control probe was used as negative control (NC). **P < 0.01. **f** Luciferase activity of Luc-CRNDE was detected by cotransfection of miRNA mimics with or without CRNDE overexpression. *P < 0.05, **P < 0.01. **g** The functional analysis of miR-181 family members was determined in NB4 cells by overexpression assays. Differentiation of NB4 cells was detected by flow cytometric analysis of the percentage of CD11b positive cells (left panel). The proliferation of NB4 cells was analyzed by CCK8 cell viability assays (right panel). The bar graph shows the average values derived from three independent experiments, and data are represented as the mean ± s.d. *P < 0.05, **P < 0.01. **h** The relationship between miR-181 and NOTCH2 was validated by CLIP-seq data from the ENCORI database. **i** The relative expression of NOTCH2 was analyzed by qRT-PCR after overexpression of miR-181 mimics in NB4 cells. *P < 0.05, **P < 0.01.

In view of CRNDE modulating cell differentiation and proliferation, we then asked the mechanism by which CRNDE regulated these functions. We first found that the CRNDE transcript was mainly located in the cytoplasm (Fig. 4a). Since cytoplasmic lncRNAs generally function as miRNA sponges to modulate downstream targets by competitively binding to miRNAs^22–24^, we hypothesized that CRNDE could act as a microRNA sponge to exert its oncogenic effects. We then applied three microRNA algorithms, miRanda^25^, DIANA-LncBase^26^, and ENCORI^27^ to predict the potential targeting microRNAs of CRNDE. As shown in Fig. 4b, the integration of these three algorithms identified 12 potential targeting microRNAs, among which 5 miRNAs were expressed in APL cells based on the miRNA expression data from the TCGA APL samples. Interestingly, 4 of these expressed miRNAs were the members of the miR-181 family, and the other was miR-136-5p (Fig. 4c). The predicted binding sites of the miR-181 family and miR-136-5p in CRNDE were shown in Fig. 4d.

To validate the interaction between predicted miRNAs and CRNDE, we performed the RNA antisense purification (RAP) experiment, in which specific CRNDE probes were used to efficiently precipitate CRNDE RNA in NB4 cells (Fig. 4e). Among these five miRNAs, only the four miR-181 family members could be coprecipitated by the CRNDE probes (Fig. 4e). The luciferase assay was further used to confirm the binding between miR-181 and CRNDE. Overexpression of miR-181 indeed repressed luciferase activity of the CRNDE reporter vector and the luciferase activity could be rescued by the ectopic expression of CRNDE (Fig. 4f).

Next, we analyzed the potential role of the miR-181 family members in APL cells. First, overexpression of these miRNA mimics promoted differentiation of NB4 cells, as detected by flow cytometry analysis (Fig. 4g). Second, the cell growth of NB4 cells was suppressed after overexpressing the miR-181c-5p or miR-181d-5p mimic, but was not observed with the miR-181a-5p and miR-181b-5p mimics (Fig. 4g). Third, to search for the downstream targets of CRNDE/miR-181 in APL cells, we used the encyclopedia of RNA interactome (ENCORI) database based on the large-scale CLIP-seq data. We found that NOTCH2, a well-known member of NOTCH signaling activated in APL development^28^, had strong binding signals with miR-181 (ClipExpNum = 28) (Fig. 4h). Indeed, we also observed that *NOTCH2* was downregulated upon CRNDE knockdown (Fig. 3d) or miR-181 overexpression (Fig. 4i). Taken together, our results indicated that CRNDE regulated cell differentiation and proliferation by directly binding to miR-181 in APL.

### CRNDE promotes leukemogenesis in *NPM1-*mutant AML

**Fig. 5:**
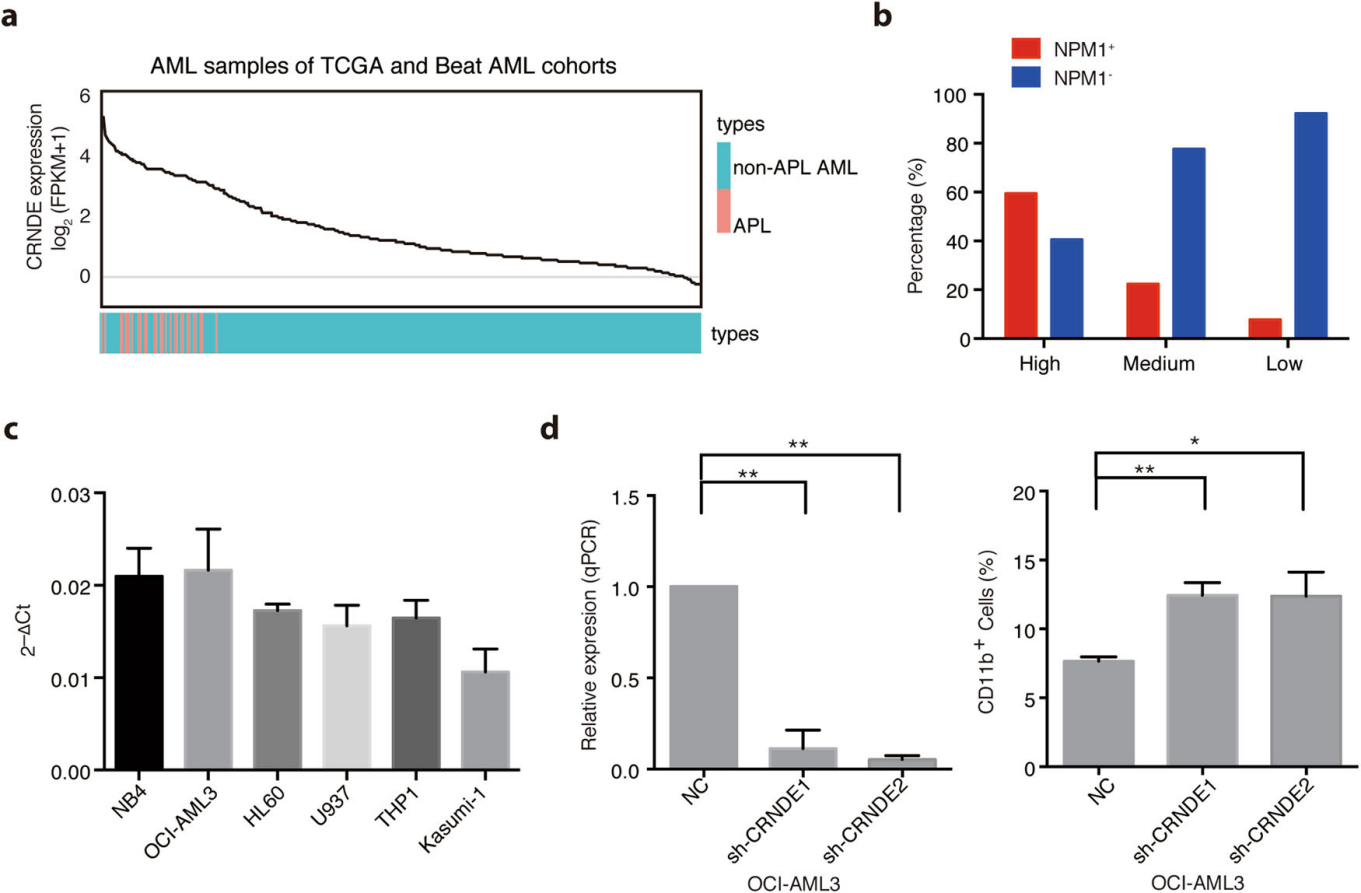
High expression of CRNDE cooperates with *NPM1* mutations to induce leukemogenesis. **a** The line chart displays the expression levels of CRNDE in 29 APL (orange) and 350 (blue) samples from the TCGA and Beat AML cohorts in order from high to low. **b** Bar graph represents the percentage of non-APL AML samples with (*NPM1*^+^) or without (*NPM1*^-^) *NPM1* mutations in groups with high, medium or low expression of CRNDE, respectively. Based on the tertile of CRNDE expression in non-APL AML samples, the patients were divided into three groups: with high expression of CRNDE (*n* = 116), medium expression of CRNDE (*n* = 116), and low expression of CRNDE (*n* = 116). **c** Relative CRNDE expression was measured by qRT-PCR assays in indicated cells. **d** The expression levels were detected by qRT-PCR in *NPM1*-mutant OCI-AML3 cells by transfection of CRNDE shRNAs (left panel). CD11b cell surface expression was detected to determine the differentiated OCI-AML3 cells (right panel). Data represent the mean of three replicates ± s.d. *P < 0.05, **P < 0.01.

Considering the oncogenic effects of CRNDE in the block of myeloid differentiation (Fig. 3b), we asked whether that CRNDE synergizes with other fusion proteins or mutations in the leukemogenesis of AML. To answer this question, we ranked CRNDE expression in 379 AML patients from the TCGA and Beat AML cohorts. As expected, all APL patients indeed had a much higher CRNDE expression (Fig. 5a). Moreover, high expression of CRNDE was also observed in some non-APL AML patients (Fig. 5a). To determine the association of CRNDE expression with the genotype status of AML in addition to APL, a distribution based on the upper (CRNDE^high^), middle (CRNDE^medium^) and the low (CRNDE^low^) tertile of CRNDE expression was used for further analysis. Interestingly, we found that high CRNDE expression was most significantly correlated with *NPM1* mutations (*P-*value <0.0001, *χ*^2^ value = 74.0546, Table 2). AML patients with *NPM1* mutations tended to be enriched in the CRNDE^high^ group (almost 60%, Fig. 5b). These suggested that CRNDE might also cooperate with the *NPM1* mutation to induce leukemogenesis. Next, we screened the expression of CRNDE in different AML cell lines by qRT-PCR assays. As shown in Fig. 5c, the high expression level of CRNDE was indeed detected in an AML cell line with the *NPM1* mutation (OCI-AML3), which was comparable to that in *PML*/*RARα*-positive NB4 cells. In contrast, CRNDE expressions in other cell lines without *NPM1* mutation were lower than that in NB4 and OCI-AML3 cells. To evaluate the role of CRNDE in *NPM1-*mutant AML, we knocked down CRNDE expression in OCI-AML3 cells. We found that CRNDE knockdown in OCI-AML3 increased the proportion of CD11b positive cells (Fig. 5d), similar to those observed in NB4 cells. These results suggested that as an oncogenic lncRNA, apart from cooperating with PML/RARα to promote the initiation of APL, CRNDE also contributed to the differentiation block in *NPM1-*mutant AML.

**Table 2:** Molecular characteristics of non-APL AML with regard to CRNDE expression.

	Non-APL AML							
	High (*n* = 116)		Medium (*n* = 116)		Low (*n* = 116)			
	Freq	%	Freq	%	Freq	%	*P* value	*χ*^2^ value
TF fusions								
* MYH11/CBFB*	2	1.72%	18	15.52%	12	10.34%	0.0231	5.1620
* RUNX1/RUNX1T1*	8	6.90%	6	5.17%	2	1.72%	0.0600	3.5378
* MLL fusions*	4	3.45%	9	7.76%	2	1.72%	0.5179	0.4180
Molecular aberrations								
* NPM1* mutation	69	59.48%	26	22.41%	9	7.76%	<0.0001*	74.0546
* FLT3-ITD*	34	29.31%	23	19.83%	19	16.38%	0.0171	5.6815
* FLT3-TKD*	13	11.21%	8	6.90%	3	2.59%	0.0096*	6.7128
* IDH1* mutation	16	13.79%	5	4.31%	6	5.17%	0.0141	6.0226
* IDH2* mutation	14	12.07%	10	8.62%	10	8.62%	0.3764	0.7823
* KRAS* mutation	4	3.45%	4	3.45%	8	6.90%	0.2099	1.5723
* NRAS* mutation	10	8.62%	17	14.66%	10	8.62%	1.0000	0.0000
* CEBPA* mutation	6	5.17%	13	11.21%	12	10.34%	0.1667	1.9124
* DNMT3A* mutation	29	25.00%	23	19.83%	16	13.79%	0.0314	4.6333
* RUNX1* mutation	3	2.59%	8	6.90%	16	13.79%	0.0014*	10.1787
* TET2* mutation	10	8.62%	8	6.90%	11	9.48%	0.8122	0.0564
* KIT* mutation	4	3.45%	7	6.03%	4	3.45%	1.0000	0.0000
* TP53* mutation	3	2.59%	6	5.17%	12	10.34%	0.0131	6.1573
* PTNP11* mutation	12	10.34%	4	3.45%	3	2.59%	0.0093*	6.7642
* WT1* mutation	14	12.07%	5	4.31%	4	3.45%	0.0082*	6.9833

*Freq* frequency. *indicates statistical significance (*P* < 0.01).

## Discussion

With efforts being put forth to the identification of oncogenic lncRNAs, the functional involvement of lncRNAs in leukemogenesis has received much attention. In this study, we found that high expression of CRNDE was associated with the development of APL and *NPM1*-mutant AML based on a large amount of clinical RNA-seq data. CRNDE knockdown induced differentiation and/or inhibited proliferation of APL cells and *NPM1*-mutant AML cells. Mechanistic investigation showed that CRNDE elicited its oncogenic effects through regulating the miR-181/NOTCH2 axis.

We determined the oncogenic role of CRNDE in the proliferation and differentiation of AML cells. CRNDE has been reported to exert oncogenic effects in diverse types of solid tumors, such as colorectal carcinoma (CRC), non-small cell lung cancer (NSCLC), glioma, pancreatic cancer and breast cancer^29–33^. Growing evidence suggests the oncogenic role of CRNDE is achieved through regulating proliferation, apoptosis, transition, invasion and migration of cancer cells^34–36^. For example, CRNDE is first identified highly expressed in CRC, and plays essential roles in promoting cell proliferation, metastasis, invasion, chemoresistance, and cellular metabolism^14,16,35,37,38^. In glioma, CRNDE can promote tumor progression by regulating cell growth, invasion, proliferation, migration, and apoptosis^15,30,39–41^. We found, in addition to promote proliferation of APL cells, CRNDE was also involved in negative regulation of myeloid differentiation. Functional analyses upon CRNDE knockdown revealed that CRNDE-regulated gene sets were enriched for genes involved in proliferation-related and myeloid differentiation-related pathways, as well as a series of myeloid markers and transcription factors associated with hematopoietic development (*CTSG*, *PRTN3*, *CEBPA*, *CEBPE*, and *NOTCH2*). Our findings provide an increased understanding of CRNDE through regulating proliferation and differentiation in leukemia development.

Mechanistic studies showed that CRNDE was located in the cytoplasm of APL cells and functioned as a miRNA sponge by directly binding miR-181. We used the in vivo RAP assay to identify miRNAs interacted with CRNDE in APL cells. This method is a more high-resolution, specific, and sensitive approach to capture the lncRNA-miRNA complexes in vivo by using a pool of antisense-specific probes^42,43^. Moreover, this in vivo assay was performed in fixed APL cells, which could better reflect the real interaction of CRNDE-miRNAs in APL cells. We identified a strong binding between CRNDE and miR-181, which was also confirmed by luciferase assays. Indeed, the miR-181 family has been reported to be involved in modulating the differentiation and proliferation of leukemia cells, which was also confirmed by our results^44,45^. Therefore, our study provides evidence that CRNDE exerts its role in regulating cell differentiation and proliferation by directly sponging miR-181.

Furthermore, *NOTCH2* is one of the downstream target genes of miR-181, and CRNDE could regulate *NOTCH2* expression by directly binding miR-181 in APL cells. Recently, several studies reveal that *NOTCH2* is highly expressed in AML and represses myeloid differentiation^46^. Furthermore, NOTCH2 is also involved in regulating cell proliferation and apoptosis^47^. More interestingly, we also found other NOTCH signaling pathway-related genes were also regulated upon the knockdown of CRNDE, such as *NOTCH1*, *MYC*, *MRPL4*, *ZWINT*, and *DDX28* (Supplementary Table S4), which is partially consistent with the previous studies^28,48^. As an oncogenic pathway, the NOTCH signaling pathway has been reported to be activated in APL and involved in modulating cell proliferation, differentiation, and cell fate in leukemia, as well as increasing self-renewal in APL mouse model^49^. Thus, our data established a novel link between CRNDE, miR-181 and NOTCH2, and further extend the function and modulating mechanism of CRNDE in leukemogenesis through the NOTCH signaling pathway.

In conclusion, our study provides new evidence that CRNDE contributes to leukemogenesis as an oncogenic lncRNA, regulating myeloid differentiation and cell proliferation in APL and *NPM1-*mutant AML. Moreover, CRNDE could promote leukemogenesis and modulate NOTCH2 level as a miRNA sponge by directly binding miR-181. Our study provides novel insight into the role of lncRNA in leukemogenesis, and provides a potential therapeutic target and biomarker for AML.

## Materials and methods

### Analysis of RNA-seq and microarray gene expression data

The RNA-seq data of AML patients were downloaded from The Cancer Genome Atlas (TCGA) Data Portal (https://tcga-data.nci.nih.gov/tcga/) (*n* = 151) and Beat AML (http://vizome.org/aml/)^50^ (*n* = 249). The GSE12662 microarray expression data set was retrieved from the Gene Expression Omnibus (GEO) Database. The detailed data analysis is available in Supplementary Methods and the code is publicly accessible on GitHub at https://github.com/rstatistics/AML-CRNDE-Analysis.

### Cell culture and reagents

AML cell lines were cultured in RPMI 1640 (Gibco, Carlsbad, CA, USA) containing 10% (vol/vol) fetal bovine serum (Moregate Biotech, Bulimba, QLD, Australia). HEK-293T cells were cultured in DMEM (Gibco) containing 10% (vol/vol) fetal bovine serum. All the cells were maintained at 37 °C in a humidified 5% CO_2_ incubator.

### Lentivirus infection and establishment of stable cell lines

Two shRNAs were designed to target CRNDE (sh-CRNDE1 and sh-CRNDE2) in NB4 or OCI-AML3 cells. Details are available in Supplementary Methods.

### miRNA transfection

The microRNA mimics were synthesized by RiboBio (Guangzhou, China). According to the manufacturer’s protocol of Nucleofector Kit V (Lonza, Cologne, Germany), NB4 cells were electrotransfected with microRNA mimics in the Amaxa Nucleofector II device (Lonza, program X-001).

### Analysis of granulocytic differentiation and cell proliferation

For flow cytometric analysis, granulocytic differentiation was assessed by detecting the percentage of CD11b positive cells. Cell proliferation was analyzed according to the manufacturer’s protocol with cell-counting kit-8 (Dojindo, Japan). Details are available in Supplementary Methods.

### Mouse studies

The mouse experiments were conducted in accordance with institutional animal protocols, provided by the Institutional Animal Care and Use Committee of Ruijin Hospital affiliated to Shanghai Jiao Tong University School of Medicine. The transplantable murine APL model was performed to analyze the Crnde functions in vivo. Details are available in Supplementary Methods.

### Isolation of nuclear and cytoplasmic RNA

Nuclear and cytoplasmic RNA were isolated and extracted using the Invitrogen™ PARIS™ Kit (ThermoFisher, USA) according to the manufacturer’s instructions. GAPDH was used as a positive control for cytoplasmic RNA fractions, and SNORA19 RNA for the nuclear fractions.

### Luciferase reporter assays

Luciferase activity was measured by the GloMax luminometer (Promega, Madison, WI, USA) using Dual-Luciferase Reporter Assay System reagents (Promega). Details are available in Supplementary Methods.

### RNA antisense purification assay

RNA antisense purification assay was performed according to the protocol previously published by Manon Torres, et al.^42^ on the Journal of Visualized Experiments (JoVE). Details are available in Supplementary Methods.

### Statistical analysis

The association between CRNDE expression and the genotype status of AML was determined by the Cochran-Armitage test for trend (Statistical Analysis System, SAS Studio). Values with *P* < 0.01 were considered statistically significant. For experiments, results are presented as arithmetic means ± s.d. of at least three independent experiments. A two-tailed Student *t*-test or Mann–Whitney test was performed to analyze the significance of the differences between two groups using GraphPad Prism 6 where appropriate. A *P*-value < 0.05 was considered significant. Kaplan–Meier survival curves for mice and *P*-value was analyzed using a log-rank (Mantel–Cox) test.

## Supplementary information

Supplementary Methods

Supplementary Figure and Table legends

Supplementary Fig. S1

Supplementary Table S1

Supplementary Table S2

Supplementary Table S3

Supplementary Table S4

Supplementary Table S5

Supplementary Table S6
