# Supplementary material for: Oncogenic role of lncRNA CRNDE in acute promyelocytic leukemia and NPM1-mutant acute myeloid leukemia
Source: Cell Death Discov. 2020 Nov 11;6:121. doi: 10.1038/s41420-020-00359-y (PMC7658230; doi:10.1038/s41420-020-00359-y)
Supplement: Supplementary file 1 — Supplementary Methods [file 41420_2020_359_MOESM1_ESM.docx]

**Supplementary Methods**

**Analysis of RNA-seq data**

The RNA-seq data of AML patients were downloaded from The Cancer Genome Atlas (TCGA) Data Portal (<https://tcga-data.nci.nih.gov/tcga/>) (n=151) and Beat AML (<http://vizome.org/aml/>)^1^ (n=249). RNA-seq reads were aligned to the GRCh38 genome using Hisat2^2^. StringTie^3^ and Ballgown^4^ were used to assemble the alignments into full and partial transcripts and estimate the expression levels of all lncRNAs. The ComBat function from sva^5^ package was used to remove batch effects. Limma algorithm^6^ was used to analyze the differential expression of the lncRNAs.

**Analysis of microarray gene expression data set**

The GSE12662 microarray expression data set was retrieved from the Gene Expression Omnibus (GEO) Database. The Robust multi-array average approach was used to normalize the raw expression data. The annotations for Affymetrix probes were implemented using the annotation data package “hgu133plus2hsensgcdf” in Bioconductor (http://www.bioconductor.org). LncRNAs were filtered based on the gene biotypes from the “EnsDb.Hsapiens.v79” annotation package. Limma package was performed to analyze differentially expressed lncRNAs between different groups.

**Cell line authentication**

All the cell lines were obtained from Shanghai Institute of Hematology. NB4 cells were authenticated by morphology and RNA-seq analysis, and were routinely tested for the existence of the PML/RARα fusion gene and their response to ATRA. OCI-AML3 cells were authenticated by the presence of the NPM1c mutation. THP1 cells were authenticated for the presence of the MLL/AF9 fusion gene. Kasumi-1 cells were authenticated for the presence of the AML1/ETO fusion gene. HL60 cells were authenticated by karyotype, and their responses to ATRA and phorbol myristic acid (PMA). U937 cells were authenticated by morphology, and ATRA or 1,25-dihydroxyvitamin D3 responsiveness. HEK-293T cells were authenticated by morphology and capability of virus production. All cell lines were mycoplasma-negative, which was routinely tested by One-step Quickcolor Mycoplasma Detection Kit (Shanghai Yise Medical Technology, Shanghai, China).

**Lentivirus infection and establishment of stable cell lines**

ShRNA targeting CRNDE (sh-CRNDE) or irrelevant nucleotide sequence (NC) were incorporated into the lentiviral vector pLVX-shRNA2 (Clontech Laboratories Inc., Palo Alto, CA, USA). The sequences of sh-CRNDE1 and sh-CRNDE2 were as follows: sh-CRNDE1, 5’-GATCCGGGGTATTCCTGTTTATAGATTCAAGAGATCTATAAACAGGAATACCCTTTTTTACGCGTG-3’, sh-CRNDE2, 5’-GATCCGGTGCTCGAGTGGTTTAAATTTCAAGAGAATTTAA

ACCACTCGAGCACTTTTTTACGCGTG-3’. The sequence of NC was 5’-GATCCAGCGTGTAGCTAGCAGAGGTTCAAGAGACCTCTGCTAGCTACACGCTTTTTTTACGCGTG-3’. 15 μg pLVX-CRNDE-shRNA (sh-CRNDE) or pLVX-NC-shRNA (NC) was cotransfected with 6 μg pMD2.G and 9 μg psPAX2 packaging constructs into HEK-293T cells to generate lentiviral particles, respectively. Viral supernatant was collected at 48 and 72 h after transfection, and used to infect NB4 or OCI-AML3 cells with adding polybrene (8 g/mL). The stable cell lines were established using the fluorescent protein ZsGreen1 as a selection marker. The infection efficiency was validated by qRT-PCR after infection for three days.

**Analysis of granulocytic differentiation**

For flow cytometric analysis, granulocytic differentiation was assessed by detecting the percentage of CD11b positive cells. Harvested 2 × 10^5^ cells were resuspended in 1×PBS, incubated for 15 min with anti-CD11b antibody (IM2581U, Beckman Coulter, Brea, CA, USA). The percentage of CD11b positive cells was analyzed for CD11b expression level using the BD LSR II flow cytometer (BD Biosciences, San Jose, CA, USA). All flow cytometric data were using the FlowJo software to analyze.

**Cell proliferation assay**

Cell proliferation was analyzed according to the manufacturer’s protocol with cell-counting kit-8 (Dojindo, Japan). Briefly, Cells (4,500 cells/well) in 100 μL RPMI-1640 medium supplemented with 10% FBS per well were seeded in sextuplicate in 96-well plate. At each time point, 10 μL CCK8 solution was added into each well and incubated at 37 °C for 3 h. The absorptions at 450 nm were determined after transfection using the microplate reader (PowerWave X; BioTek, Winooski, VT, USA).

**Mouse experiments**

The APL transplantable model was generated by injecting APL blasts isolated from bone marrows of hMRP8-PML/RARα transgenic APL mice according to previous study^7^. The FVB/NJ recipient mice randomized into two groups: control group and si-CRNDE group (n=6 for each group). The APL blast cells (1×10^5^ per mouse) were electrotransfected with murine Crnde or irrelevant control siRNA, and then injected intravenously into recipient mice (male, 6-8 week old). The peripheral blood from the mouse tail vein were isolated and removed red blood cells by Red Blood Cell Lysis Buffer (Sangon Biotech, China) for further investigation 19 days after transplantation. The percentage of GFP-positive APL blasts was assessed by flow cytometry and the white blood cell counts were counted by pocH-100iV Diff hematology analyzer (Sysmex Corporation, Kobe, Japan). All mice developed leukemia about 20 to 30 days after transplantation. The si-Crnde sequence was as follows: 5’-CCUCAGAGCUGCAGACAAATT-3’(sense). For mouse studies, no blinding was done.

**RNA extraction, reverse transcription, and qRT-PCR analysis**

RNA was extracted using an RNeasy Mini Kit (QIAGEN, Hilden, Germany) according to the protocol. RNA was reverse transcribed into cDNA using PrimeScript™ RT reagent Kit with gDNA Eraser (Takara, Japan). Real-time quantitative PCR was performed using ChamQ Universal SYBR qPCR Master Mix (Vazyme, Nanjing, China) in the Vii7 Real-Time PCR System (Applied Biosystems Inc., Foster City, CA, USA). GAPDH was selected as an endogenous control. The specific qRT-PCR primers are listed in **Supplementary Table S6**.

**RNA Sequencing and differentially expressed genes screening**

Total RNA was extracted from retroviral transfected NB4 cells, including a negative control group and sh-CRNDE group using the RNeasy mini kit (Qiagen, Chatsworth, CA, USA) according to the manufacturer’s protocol. TruSeq RNA Sample Prep Kit v2 (San Diego, CA, USA) was used for library preparation, and enriched cDNA libraries were sequenced using an Illumina Miseq sequencer instrument. The differentially expressed genes before and after CRNDE knockdown were analyzed as previous described^8^. The RNA-seq data of NB4 cells before and after CRNDE knockdown generated in this manuscript are available at NCBI GEO under accession number GSE148489.

**miRNA prediction**

Three software algorithms miRanda (<http://www.microrna.org/>), DIANA-LncBase v2 ([http://carolina.imis.athena-innovation.gr](http://carolina.imis.athena-innovation.gr/)) and ENCORI ([http://starbase.sysu.edu.cn](http://starbase.sysu.edu.cn/)) were used to predict the potential miRNAs targeted by CRNDE.

**Plasmid construction and luciferase reporter assays**

The empty pLuc and pcDNA3.0-GAPDH plasmids used in the luciferase assays were obtained from Prof. Shenglin Huang at Fudan University^9^. The fragment of the CRNDE sequence was ligated into pLuc and pcDNA3.0 to construct pLuc-CRNDE and pcDNA-CRNDE plasmids, respectively.

HEK-293T cells were seeded at a density of 8 × 10^4^ cells per well in 24-well plates for 24 h before transfection. A total of 200 ng pLuc-CRNDE, 20 ng pRL-SV40 luciferase plasmid, 800 ng pcDNA-CRNDE or pcDNA-GAPDH, and microRNA mimics (or negative control) at the indicated concentration were transiently transfected into HEK-293T cells using Lipofectamine 2000 (Invitrogen, Carlsbad, CA, USA). After 24 h of transfection, luciferase activity was measured with GloMax luminometer (Promega, Madison, WI, USA) using Dual-Luciferase Reporter Assay System reagents (Promega) according to the manufacturer’s protocol. The pRL-SV40 luciferase plasmid was used as an internal control for transfection efficiency. The negative control microRNA was used for calculating the relative luciferase activity of each target microRNA.

**RNA antisense purification assay**

RNA antisense purification assay was performed according to the protocol previously published by *Manon Torres*, *et al.*^10^ on the Journal of Visualized Experiments (JoVE). Briefly, NB4 cells (5 × 10^7^ per group) were fixed with 1% paraformaldehyde for 10 min and quenched the paraformaldehyde action by adding glycine (1.25 M) for 5 min. The cells were washed with PBS twice and resuspended in the lysis buffer (50 mM Tris-HCl (pH 7.0), 10 mM EDTA, 1% SDS with 200 U/mL of an RNase inhibitor (Vazyme Biotech, China) and an EDTA-free protease inhibitor cocktail (Sigma-Aldrich)). After sonication on a Bioruptor Pico (5 series of 30 s On and 30 s Off), the lysate was mixed with a 2 volume of hybridization buffer (750 mM NaCl, 1% SDS, 50 mM Tris-HCl (pH 7.0), 1 mM EDTA, 15% formamide added extemporaneously) containing 500 pmol biotinylated probes and incubated at room temperature for 4 h with rotation. Dynabeads (Dynabeads M-280 Streptavidin, Thermo Fisher Scientific, Waltham, MA, USA) were mixed into the lysate and incubated at room temperature overnight with rotation. RNA complex and beads were washed with wash buffer (SDS 0.5%, SSC 2×) five times. After proteinase K incubation for 45 min at 50 °C, and then 10 min at 95 °C, eluted samples were resuspended in Trizol reagent to extract RNA products. The coprecipitated miRNAs were determined by Bulge-Loop^TM^ miRNA qRT-PCR (RiboBio, Guangzhou, China). The sequences of biotin-labeled probes from 5’ to 3’ for CRNDE pull down are as following: CTCCGCCTCGCTTAGACATT, GACGACTCGGGCCTACTGCG, ACACTTAACACCTCTCCTCC, AGACCAGCCTTGGGATGAAT, TCCTCCTTCCAATAGCCAGT, TAGCACTCACAATGAGTCAT, ATTTCAAAGACCAACGGCTG, GTCTATAAACAGGAATACCC, CTAAGCATTTTCAGTAAAAC.

**References**

1. Tyner, J. W. et al. Functional genomic landscape of acute myeloid leukaemia. *Nature* **562,** 526-531 (2018).

2. Kim, D., Langmead, B. & Salzberg, S. L. HISAT: a fast spliced aligner with low memory requirements. *Nat. Methods* **12,** 357-360 (2015).

3. Pertea, M., Pertea, G. M., Antonescu, C. M., Chang, T. C., Mendell, J. T. & Salzberg, S. L. StringTie enables improved reconstruction of a transcriptome from RNA-seq reads. *Nat. Biotechnol.* **33,** 290-295 (2015).

4. Frazee, A. C., Pertea, G., Jaffe, A. E., Langmead, B., Salzberg, S. L. & Leek, J. T. Ballgown bridges the gap between transcriptome assembly and expression analysis. *Nat. Biotechnol.* **33,** 243-246 (2015).

5. Leek, J. T., Johnson, W. E., Parker, H. S., Jaffe, A. E. & Storey, J. D. The sva package for removing batch effects and other unwanted variation in high-throughput experiments. *Bioinformatics* **28,** 882-883 (2012).

6. Ritchie, M. E. et al. limma powers differential expression analyses for RNA-sequencing and microarray studies. *Nucleic Acids Res.* **43,** e47 (2015).

7. Lin, J. et al. TRIB3 Stabilizes High TWIST1 Expression to Promote Rapid APL Progression and ATRA Resistance. *Clin. Cancer Res.* **25,** 6228-6242 (2019).

8. Pertea, M., Kim, D., Pertea, G. M., Leek, J. T. & Salzberg, S. L. Transcript-level expression analysis of RNA-seq experiments with HISAT, StringTie and Ballgown. *Nat. Protoc.* **11,** 1650-1667 (2016).

9. Zheng, Q. et al. Circular RNA profiling reveals an abundant circHIPK3 that regulates cell growth by sponging multiple miRNAs. *Nat. Commun.* **7,** 11215 (2016).

10. Torres, M. et al. RNA pull-down procedure to identify RNA targets of a long non-coding RNA. *J. Vis. Exp.***,** e57379 (2018).
