# Supplementary material for: Oncogenic role of lncRNA CRNDE in acute promyelocytic leukemia and NPM1-mutant acute myeloid leukemia
Source: Cell Death Discov. 2020 Nov 11;6:121. doi: 10.1038/s41420-020-00359-y (PMC7658230; doi:10.1038/s41420-020-00359-y)
Supplement: Supplementary file 2 — Supplementary Figure and Table legends [file 41420_2020_359_MOESM2_ESM.docx]

**Supplementary Figure and Table legends**

**Supplementary Fig. S1 CRNDE-g is the most abundant transcript in NB4 cells.**

**a** Schematic representation of alternative splicing transcripts from the *CRNDE* locus (minus strand) from NCBI AceView database. Plotting scale and nucleotide number (top) is for chromosome 16, hg38. The red boxes, lines and arrows represent exons, introns and transcription direction, respectively. **b** The relative expression levels of different CRNDE transcripts in NB4 cells were detected using qRT-PCR with specific primers. The *y*-axis represents the mean 2^–ΔCt^ values from three independent experiments, and error bars shows the standard deviation.

**Supplementary Table S1: Differentially expressed lncRNAs in APL versus non-APL AML samples.**

**Supplementary Table S2: Differentially expressed lncRNAs in APL versus normal bone marrows.**

**Supplementary Table S3: The pathogenesis-related lncRNAs in APL.**

**Supplementary Table S4: Differentially expressed genes after CRNDE knockdown.**

**Supplementary Table S5: IPA biofunctions analysis for the differentially expressed genes by CRNDE knockdown.**

**Supplementary Table S6: The primer pairs for qRT-PCR.**
