# Supplementary figures and images for: Oncogenic role of lncRNA CRNDE in acute promyelocytic leukemia and NPM1-mutant acute myeloid leukemia
Source: Cell Death Discov. 2020 Nov 11;6:121. doi: 10.1038/s41420-020-00359-y (PMC7658230; doi:10.1038/s41420-020-00359-y)

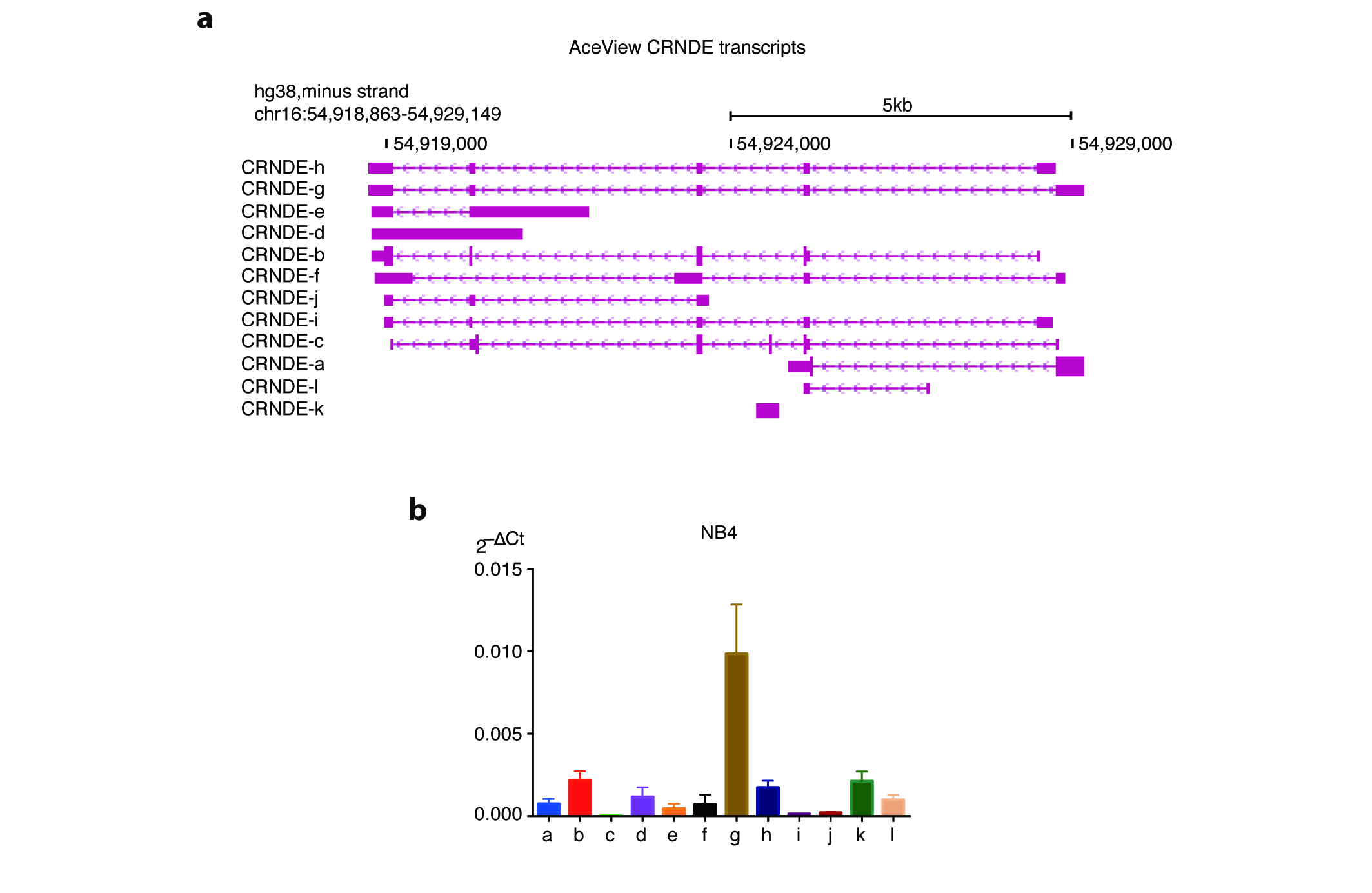

Supplement: Supplementary file 3 — Supplementary Fig. S1 [file 41420_2020_359_MOESM3_ESM.tif]
